# Pneumonia

**Published:** 1885-10

**Authors:** 


### PNEUMONIA.

Aconite.—Chill, followed by intense fever, hot, dry skin, quick and hard pulse; accelerated, labored, incomplete respiration, with restlessness, palpitation, fear of death, dry cough, soreness and heat in chest; later, burning-shooting or burning-pressing pains in chest, with painfulness to external pressure; oppression and acceleration of respiration, sense of weariness and exhaustion in chest; hyperæmia of lungs, sputa thin, frothy, tinged with blood.

Arnica.—Caused by mechanical injury and where in plethoric persons pneumonic infiltration shows a tendency to hæmorrhage; dry cough, shaking the whole body, with tough, bloody sputa.

Arsenic.—Extreme prostration, clammy sweat, great thirst, drinking little and often; shortness of breath on slight exertion; dry and dark tongue and lips, diarrhœa; singing and buzzing in ears; tendency to colliquation and dissolution; threatened gangrene, with ichorous expectoration, fetid or dingy green (Chin., Lach.). In sudden œdema, with passive hyperæmia of the lungs (sometimes caused by defects of the right side of the heart); in old people, from repercussed eruptions; in asthmatic persons; hypostatic pneumonia; pneumonia notha in old people, with danger of paralysis of lungs; hoarse after midnight, sudamina; *very restless;* worse after midnight.

Bellad.—Cerebral complication, with great nervousness, intense and constant delirium; restlessness, sleepiness, but cannot sleep; picking at bedclothes; flushed face; congested eyes; pneumonia arising from or accompanying acute bronchitis; pneumonia of drunkards (Nux v.) and of old people; pneumonia of a typhoid character from the beginning.

Bromium.—Hepatization of lower lobes; right lung mostly affected; sensation of weakness and exhaustion in the chest; sensation of constriction impedes respiration, with dry, tickling cough; loose cough night and day, but no expectoration.

Bryonia.—Lobular pneumonia, anxiety from oppressed inspiration, pressure on middle or lower part of sternum; bruised feeling in chest; shooting pains in chest; red hepatization and cough, but expectoration not yet free, sputa viscid, tenacious, of a brickdust color; foul tongue, constipation; gastric catarrh; thirst for large quantities; abdominal breathing; *inclination to lie perfectly still.*

Cactus gr.—Oppression of respiration, pricking pains; acute intense pains with the cough; bloody sputa; hard, quick vibrating pulse; feeling of constriction in chest preventing free speech; sharp wandering pains in chest, especially in scapular region; cough, with thick yellow sputa like boiled starch.

CARBO VEG.—Profuse cool perspiration, pulse small and rapid; great prostration; tongue dry, with little or no thirst; foul, decaying diarrhœic stools; breath foul, craves cold air; foulness of all secretions; rattling in chest; distressing cough, without any expectoration, by spells, or fetid, gangrenous sputa. Paralysis of lungs; pneumonia complicated with affections of right heart, or, in emphysematous patients, with old bronchial catarrhs.

CHELID.—Shortness and difficulty of breathing, with tightness and anxiety of the chest, violent stitches in right lung going to the lower edge of right shoulder-blade; short, dry cough, which increases the pain; great and quite irregular palpitation of heart; short and quick breathing, with anxiety, as if he must choke; bilious pneumonia.

CHINA.—Hectic symptoms, with marked prostration, from loss of blood; pneumonia complicated with hyperæmia of liver, icterus, intestinal catarrh; incipient gangrene; hæmoptysis, with subsequent suppuration of lungs and stitches in chest, worse during deep breathing and sudden movements.

CUPRUM.—Lobular pneumonia, when formation of abscess threatens; beginning paralysis of lungs, indicated by sudden difficulty of breathing, followed by great prostration; complication with whooping-cough; face earthy, dirty, bluish; roof of mouth red; sweat sour-smelling; diarrhœa.

GELSEMIUM.—Congestive pneumonia, with suffering under the scapulæ, both sides, caused by checked sweat; short paroxysms of pain in superior part of right lung, on taking a deep breath; rawness and soreness of chest; slow, heavy breathing; pulse slow, full; thirstlessness.

HEPAR.—Mild suppurative stage, extending only over small part of a lung, with lentescent fever; chronic pneumonia, with profuse purulent expectoration; weakness of the chest, preventing talking.

HYOS.—Pneumonia, with cerebral symptoms (Bell.), delirium, sopor; dry, fatiguing nightcough, or rattling in chest; pneumonia complicated with typus; hypostatic pneumonia in the course of other chronic affections; pneumonia senilis, with acute œdema of lungs; pneumonia of drunkards.

IODINE.—*Pneumonia crouposa;* tendency to bronchial and pulmonary congestion and hæmorrhage; sensation of weakness in chest, with anxiety and oppression, and burning, tearing, stabbing pains; sensation, as if something resisted the expansion of the chest; cough, with dyspnœa and blood-streaked expectoration. Also during third stage, where slow suppuration sets in without marked febrile symptoms in tuberculous patient, and causes a slowly progressing hectic condition, entirely confined to lungs.

IPECAC.—*Infantile pneumonia;* respiration rapid, difficult, surface blue, face pale; rattling of large bubbles, or fine rattling noises in chest, with spasmodic cough and nausea; hyperæmia of brain, without sopor; convulsions.

KALI BICH.—*Pneumonia crouposa,* with expectoration of tough stringy mucus; coughs up casts of elastic fibrinous nature; loud mucous râles; *pains from back to sternum,* or from midsternum darting to between the shoulders; morning aggravation.

KALI CARB.—*Infantile pneumonia;* during whooping-cough; great dyspnœa, preventing the child from sleeping or drinking; stitches in chest; difficulty of raising the mucus, although constantly coughing; wheezing and rattling breathing, choking cough; inability to breathe deeply; pneumonia, with stitches through right chest, hepatization of right lung, worse when lying on right side; abscess of lung, with expectoration of pus and blood.

KALI IOD.—Pneumonia in the beginning when the disease localizes itself; also with so extensive hepatization as to cause cerebral congestion and serous exudation; face red, pupils large, urine suppressed, one side as if paralyzed; cough dry, hawking, later copious green sputa; œdema pulmonum, with pneumonia.

KREOS.—*Gangrene of lungs;* dry wheezing cough; after every coughing spell copious, purulent expectoration; difficult breathing, with anxiety; sensation of oppression in chest, better from pressure.

LACHESIS.—Pneumonia, with hepatization, mostly of left lung, and great dyspnœa on awaking; especially useful in removing deposits resulting from inflammations in lungs already invaded by tubercles, or from low-graded chronic inflammations, developing during the progress of other diseases; suffocation and shortness of breath from the cough; frothy expectoration, mixed with blood; purulent dissolution of exudation during third stage; threatened gangrene of lungs, with fetid breath and sputa.

LACHNAN.—*Typhoid pneumonia;* hot and oppressed feeling in the lungs and heart, with dizziness; cough worse in bed, preventing sleep; stitches following one another in quick succession, while at rest and when moving; unnatural brightness of eyes, with red flushed face.

Lyc.—*Typhoid or neglected pneumonia* after suppressed menses, with continuing hepatization and purulent sputa; adynamia and night-sweats as sequelæ of neglected pneumonia; or, pneumonia, with raising of a mouthful of mucus at a time, of a light-rust color, stringy, and easily separated; constant tickling cough, worse at night; numerous loud mucous râles, with rare and scanty sputa; cough loose, full and deep, sounding as if the whole parenchyma of the lung were softened; circumscribed redness of face; *fanlike motion of nostrils.*

Mercurius.—Pneumonia and bronchitis, especially when the patients are disposed to blennorrhœa, or have a profuse expectoration of viscid bloody mucus; *bilious pneumonia*, with great tenderness over the right hypochondrium; *asthenic pneumonia*, with feeling of weight in lungs, short cough, and expectoration of bloody saliva; *epidemic broncho-pneumonia*, with deep irritation of the nervous system; nose, larynx, and trachea become suddenly dry, dyspnœa sets in with spasmodic cough, worse at night, and yellow-green, blood-streaked expectoration; skin burning hot, at times covered with copious sweat; tongue yellow, soon becomes dry; senses dull, violent headache, soporous condition, with light delirium; complains of little or no pain (influenza).

Natrum sulph.—*Sycotic pneumonia;* inexpressible agony; slowly coagulated blood; stitching pains running up from abdomen to left chest; dry cough, with soreness in chest, rough feeling in throat, particularly at night; had to sit up and hold chest with both hands; loose purulent sputa in the morning.

Nitric acid.—*Pneumonia of old and cachectic people;* sputa are raised with difficulty; awakens often all stopped up with mucus, and must expectorate before he can breathe more easily; sputa of blood mixed with clots during the day; pulse intermits.

Nux vom.—*Broncho-pneumonia*, especially of drunkards, or of persons suffering from piles.

Opium.—*Infantile pneumonia*, where the pulmonary inflammation is disguised by symptoms of cerebral congestion and oppression; cyanotic color of the upper part of body, with slow stertorous respiration; difficult intermitting breathing, as from paralysis of lungs; blood thick, frothy, mixed with mucus; great oppression, burning about heart, tremor, feeble voice; anxious sleep, with starts; legs cold, chest hot.

PHOSPHORUS.—*Broncho-pneumonia;* dryness of air passages; excoriated feeling in upper chest; great weight on chest or tightness; chest sore, bruised; hepatization of lower half of right lung; dulness of sound on percussion; bronchial respiration, frequently attended with crepitation and rattling. *Typhoid pneumonia,* not a genuine inflammation, rather an accumulation of blood in the veins, and extravasation of fluid blood in the tissues of the organ; the patient is weak, with feeble pulse, sighs occasionally, is unable to use his lungs, not from pain, but merely from weakness and hyperæmic stagnation; pulse thready; cold sweat; *pleuro-pneumonia,* with extensive implication of the pleura; hepatization, with mucus or bloody sputa; coughing increases the difficulty of breathing; during the third stage purulent infiltration of the parenchyma, with mental depression, slight delirium, carphologia and subsultus tendinum, rapid prostration, cold clammy sweat, small, feeble, frequent pulse, dim eyes, sunken features, dry lips and tongue, short, laborious breathing, oppression and anxiety, tedious cough and expectoration, involuntary diarrhœa; threatened paralysis of lungs; tuberculosis in tall, slender, weak-chested persons.

RANUNCULUS BULB.—Bright-red checks, with clean tongue; short and very oppressed breathing, with scarcely audible respiratory murmurs; dry heat; prostration from the start; small, very rapid pulse, with great vascular and cardiac excitement, nausea, and even faintness on motion.

RHUS TOX.—*Typhoid pneumonia,* often from resorption of pus, with tearing cough and restlessness, as rest aggravates the pain and dyspnœa; tongue red at tip; loss of strength, sopor, hardness of hearing, unconscious defecation and urination, dryness and heat of skin, dry and sooty tongue; dyspnœa worse from distention of pit of stomach; sputa bloody or of color of brickdust, or green cold mucus, of putrid smell.

SANGUIN.—Great difficulty of breathing, lies upon back, with head elevated; not much pain in chest, but that of a stitching-burning character; pulse small and quick; face and extremities inclined to be cold, or hands and feet burning, with circumscribed redness and burning heat of the cheeks, especially after noon; cough, with tough and rust-colored sputa, or in third stage purulent and offensive; diarrhœa, night-sweats.

SILICEA.—Chronic neglected pneumonia, passing over into suppuration; dyspnœa when lying on back or coughing; lungs feel sore; excruciating, deepseated pains in lungs; sputa profuse, fetid, green, and purulent, often tastes greasy.

Spongia.—*Broncho and croupous pneumonia;* sputa tastes sour or salty, worse when lying down; wheezing, anxious breathing; burning and soreness in chest; during the stage of resolution with profuse secretion and expectoration of mucus, inability to lie down; the cough relieved by eating and drinking (Caust.).

Squilla.—Suitable in pneumonia or pleurisy after bleeding, or when accompanied with gastric symptoms; pain in chest worse mornings, also cough; sputa copious and thin.

Sulphur.—*Pneumonia assumes a torpid character, with slow solidification of the lungs;* there may still be much rattling of phlegm in chest; frequent weak, faint spells, and flashes of heat; feels suffocated, wants doors and windows open; constant heat on top of head. *Torpid typhoid pneumonia,* with short rapid breathing, a mere heaving of the chest; cough and expectoration nearly impossible; the patient responds sluggishly, comprehends slowly; worse about midnight. *Neglected pneumonia* occurring in psoric patients, and which threatens to terminate in tuberculosis pulmonum, or in phthisis pituitosa. Pneumonia passing through its first stages normally and then remains stationary; such a deficiency of reaction points to Sulphur as the remedy, where it accomplishes the absorption of the infiltration and prevents suppuration.

Tartar emetic.—*Pneumonia catarrhalis;* paroxysms of cough, with suffocative arrest of breathing; rattling hollow cough; cough, with heat and moist hands, sweat about the forehead; anxious oppression of chest, with rising of heat, reaching as far as the heart; dyspnœa, with desire to cough and a quantity of rattling mucus in the chest; *œdema pulmonum;* impending paralysis of lungs; cyanosis; suitable especially to infants and old people.

Veratrum album.—Dyspnœa, with rattling of mucus; fear of suffocation; frothy serous sputa; blue face; dry and spasmodic cough, accompanied by marked cerebral congestion; hurried and small pulse, cold skin and *cold sweat,* with excessive debility; capillary bronchitis, œdema of lungs; suitable often to old people.

Veratrum viride.—Pneumonia; pulse hard, strong, quick; engorgement of lungs; sputa containing large masses of blood, with faint feeling in stomach, nausea, slow and intermittent pulse; constant burning distress in cardiac region; heart beats loud, strong; *great arterial excitement;* great cerebral congestion.

